# Association of sarcopenia with survival in advanced NSCLC patients receiving concurrent immunotherapy and chemotherapy

**DOI:** 10.3389/fonc.2022.986236

**Published:** 2022-09-23

**Authors:** Fabian J. Bolte, Sloane McTavish, Nathan Wakefield, Lindsey Shantzer, Caroline Hubbard, Arun Krishnaraj, Wendy Novicoff, Ryan D. Gentzler, Richard D. Hall

**Affiliations:** ^1^ Department of Medicine, University of Virginia, Charlottesville, VA, United States; ^2^ School of Medicine, University of Virginia, Charlottesville, VA, United States; ^3^ Department of Medicine, Division of Hematology and Oncology, University of Virginia Cancer Center, Charlottesville, VA, United States; ^4^ Department of Radiology and Medical Imaging, University of Virginia, Charlottesville, VA, United States; ^5^ Department of Public Health Sciences and Orthopedic Surgery, University of Virginia, Charlottesville, VA, United States

**Keywords:** metastatic lung cancer, non-small cell lung cancer, immunotherapy, sarcopenia, overall survival, body mass index, performance status

## Abstract

**Background:**

Frailty, sarcopenia and malnutrition are powerful predictors of clinical outcomes that are not routinely measured in patients with non-small cell lung cancer (NSCLC). The primary aim of this study was to investigate the association of sarcopenia, determined by the psoas muscle index (PMI) with overall survival (OS) in patients with advanced NSCLC treated with concurrent immune checkpoint inhibitor (ICI) and chemotherapy (CTX).

**Methods:**

We retrospectively reviewed data from a cohort of patients with locally advanced or metastatic NSCLC who were treated between 2015 and 2021 at the University of Virginia Medical Center. The cross-sectional area of the psoas muscle was assessed on CT or PET/CT imaging prior to treatment initiation. Multivariate analysis was performed using Cox proportional hazards regression models.

**Results:**

A total of 92 patients (median age: 64 years, range 36-89 years), 48 (52.2%) men and 44 (47.8%) women, were included in the study. The median follow-up was 29.6 months. The median OS was 17.8 months. Sarcopenia, defined by a PMI below the 25th percentile, was associated with significantly lower OS (9.1 months in sarcopenic patients vs. 22.3 months in non-sarcopenic patients, P = 0.002). Multivariate analysis revealed that sarcopenia (HR 2.12, P = 0.0209), ECOG ≥ 2 (HR 2.88, P = 0.0027), prognostic nutritional index (HR 3.02, P = 0.0034) and the absence of immune related adverse events (HR 2.04, P = 0.0185) were independently associated with inferior OS.

**Conclusions:**

Sarcopenia is independently associated with poor OS in patients with advanced NSCLC undergoing concurrent ICI and CTX.

## Introduction

Frailty is a state of increased vulnerability to stressors due to a decline in function and reserves across multiple physiologic systems (1, 2). In large prospective cohort studies, it has been shown that frailty is directly related to poor health outcomes including hospitalizations, therapy-related complications, disability, and mortality (3, 4). Sarcopenia, defined as a loss of muscle mass and function, constitutes an important component of physical frailty (5). The prevalence of sarcopenia among cancer patients is considerably high (6). In a systematic review and meta-analysis including 13 studies with 1810 patients, the pooled prevalence of sarcopenia was 43% in patients with NSCLC and 52% in patients with SCLC (7). Additionally, sarcopenia has been shown to be an independent predictor of reduced survival for patients with different stages of NSCLC (7, 8), which has been attributed to chemotherapy-induced toxicity and post-operative complications (9). However, treatment strategies have changed, and little is known about whether or how sarcopenia affects outcomes of NSCLC patients undergoing concurrent ICI and CTX. A more recent review and meta-analysis on the prognostic value of sarcopenia in advanced lung cancer patients receiving immune checkpoint inhibitor therapy highlights the need for additional research to better elucidate the prognostic value of sarcopenia and its association with functional impairment and treatment-related toxicity (10).

The prognosis of patients with advanced lung cell cancer is dependent on patient- and disease-specific factors. The former commonly includes age, sex, body mass index (BMI) and performance status. However, sarcopenia is not routinely considered in the assessment due to lack of standardization. The European Working Group on Sarcopenia in Older People (EWGSOP) proposed a muscle mass of two standard deviations below healthy adults as an operational definition of sarcopenia and various technologies including Dual x-ray absorptiometry (DXA), computed tomography (CT), magnetic resonance imaging (MRI), and bio-impedance analysis (BIA) have been recommended for evaluation of muscle mass (11–13). In previous studies of patients with biliary malignancies (11, 14), pancreatic cancer (15), esophageal cancer (16) and colorectal cancer (17–19) sarcopenia was defined by the psoas muscle index (PMI) obtained from secondary analysis of cross-sectional abdominal imaging.

We performed a retrospective analysis of the PMI and intramuscular adipose content (IMAC) in a cohort of patients with locally advanced or metastatic NSCLC. The primary aim of this study was to investigate if sarcopenia, determined by the PMI, is associated with overall survival (OS) in a cohort of patients with advanced non-small cell lung cancer (NSCLC) treated with concurrent immune checkpoint inhibitor (ICI) and chemotherapy (CTX). Our secondary aim was to study the association between PMI and Eastern Cooperative Oncology Group (ECOG) performance status as well as BMI.

## Materials and methods

### Patient eligibility and data collection

We conducted a retrospective study including 92 patients with histologically confirmed locally advanced NSCLC not amenable to definitive chemotherapy and radiation or metastatic NSCLC who were treated at the University of Virginia Medical Center between 2015 and 2021 with concurrent ICI and CTX. Patients without cross-sectional abdominal imaging (CT or PET/CT) within 120 days of treatment initiation and patients with lumbar artefacts such as prior lumbar fusion surgery not amenable to psoas muscle analysis were excluded from the study. The following data was collected at the time of initiation of concurrent ICI and CTX: Age, sex, race (White/Caucasian, Black/African American, other), height, weight, ECOG performance status, smoking status (former, current, never), lung cancer histology (adenocarcinoma, squamous cell cancer, or other), PDL-1 status (positive >= 1%, negative < 1%), lung cancer stage (locally advanced, metastatic), brain metastasis (no, yes), absolute lymphocyte count and serum albumin. The occurrence of immune-related adverse events (irAE) was assessed retrospectively from documentation in the electronic medical record, and they were graded according to the Common Terminology Criteria for Adverse Events (CTCAE Versions 4.03 and 5.0). The neutrophil-to-lymphocyte ratio (NLR) was calculated as absolute neutrophil count (10^3^/μL)/absolute lymphocyte count (10^3^/μL). The prognostic nutritional index (PNI) was calculated as 10 x serum albumin (g/dL) + 0.005 x absolute lymphocyte count (10^3^/μL). The NLR and PNI were calculated based on laboratory studies prior to the initiation of ICI and CTX. Programmed death ligand 1 (PDL-1) positive tumors were defined by PD-L1 expression ≥ 1% or more of tumor cells based on a PD-L1 immunohistochemistry assay, Ventana SP263 on Leica Bond III performed as a lab-developed test and validated against Dako 22C3 for concordance. OS was defined as the number of months alive after treatment initiation.

### Image analysis

**Figure 1:**
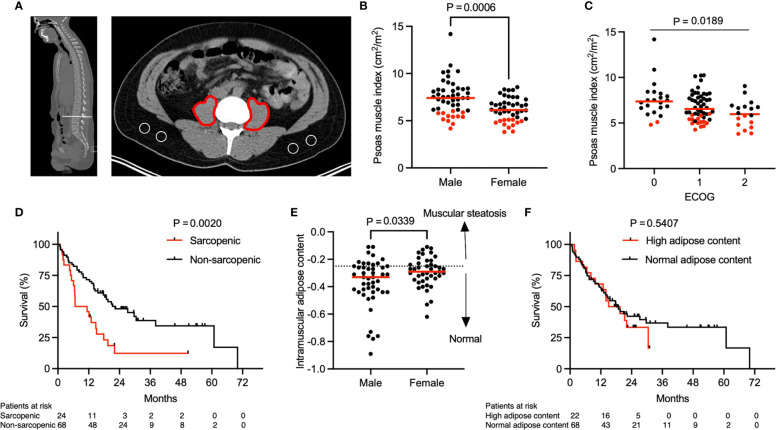
Association of sarcopenia with overall survival. Representative **(A)** sagittal and transversal computed tomography (CT) images at the inferior aspect of the third lumbar vertebra. Red markings indicate the cross-sectional area of both psoas muscles and areas encircled in white indicate subcutaneous fat. **(B)** Psoas muscle index (PMI) stratified by gender (n = 92). P value calculated using the Mann-Whitney U test. **(C)** Patient ECOG score in relation to PMI (n = 92). P value calculated using the Kruskal-Wallis test. **(D)** Kaplan-Meier curve depicting survival analysis based on sarcopenia. **(E)** Intramuscular adipose content (IMAC) stratified by gender (n = 90). Two patients were excluded from analysis due to almost complete absence of subcutaneous fat tissue on abdominal imaging. Dotted line represents the threshold between normal and increased intramuscular adipose content based on sex-specific 25^th^ percentile. P value calculated using the Mann-Whitney U test. **(F)** Kaplan-Meier curve depicting survival analysis based on IMAC. P values in **(D, F)** were calculated with the log-rank test. Each tick mark on the Kaplan-Meier curve represents a censored event. Each data point in **(B, C, E)** represents an individual patient. Red dots in **(B, C)** indicate sarcopenic patients with a PMI value below the sex-specific 25^th^ percentile and cut offs were 6.03 cm^2^/m^2^ for males and 5.11 cm^2^/m^2^ for females. Red lines represent the median of the dataset.

The cross-sectional area of the psoas muscle and the muscle attenuation were assessed by secondary analysis of CT or PET/CT images, which had been taken for diagnostic purposes with a median of 27 days (IQR 8 to 36) prior to initiation of therapy. The inferior aspect of the third lumbar vertebra on cross-sectional abdominal imaging was chosen as a standard landmark to measure the psoas muscle area and muscle attenuation. The psoas muscle was identified and quantified by use of Hounsfield unit (HU) thresholds (-310 to +390). The psoas muscle measurements as indicated in **Figure 1A** have been performed by an investigator using Philips Vue PACS v12.2.6.2010001 who was trained individually by a physician certified in diagnostic radiology. These investigators were blinded to the clinical outcome. The PMI was calculated as cross-sectional area of both psoas muscles (cm^2^) divided by height^2^ (m^2^) as previously described (20). Patients were classified as sarcopenic and non-sarcopenic based on the sex specific 25^th^ percentile of the PMI in our patient cohort. The intramuscular adipose content (IMAC) was determined by the ratio of the attenuation in HU of the psoas muscle and subcutaneous fat. The ratios were expressed as negative numerical values because the attenuation of subcutaneous fat is negative around -100 HU.

### Statistical analysis

Data is shown as median with interquartile range (IQR). Differences between groups of patients were studied by use of Fisher’s exact test, Pearson’s χ2 test, Mann-Whitney U test and Kruskal-Wallis test. All tests were two sided and significance was reported at the p < 0.05 level. Spearman correlation was used to measure the degree of association between two variables. OS was analyzed using the Kaplan-Meier method and differences between groups were compared with the log-rank test. Multivariate analysis was performed using Cox proportional hazards regression models. Variables known to affect survival in patients with advanced lung cancer were included into a multivariate Cox proportions hazards model, and 95% confidence intervals (CI) for the estimated hazard ratios (HR) were calculated. Statistical analysis was performed with GraphPad Prism version 9.0 (GraphPad Software Inc., San Diego, CA).

## Results

### Baseline characteristics of patients classified by PMI as sarcopenic and non-sarcopenic

A total of 92 patients (median age: 64 years, range 36-89 years), 48 (52.2%) men and 44 (47.8%) women, were included in the study. All patients had locally advanced (n = 9, 9.8%) or metastatic (n = 83, 90.2%) NSCLC. The cross-sectional area of the psoas muscle and the muscle attenuation were assessed on CT or PET/CT imaging obtained prior to treatment initiation (median 27 days, IQR 8 to 36 days). The PMI, defined by the bilateral psoas muscle area in relation to the height of the patient, differed significantly between males and females (**Figure 1B**, median, 7.41 cm^2^/m^2^ for men and 6.13 cm^2^/m^2^ for women, P = 0.0006). Thus, in our patient cohort sarcopenia was defined as a PMI below the 25^th^ sex specific percentile and cut offs were 6.03 cm^2^/m^2^ for men and 5.11 cm^2^/m^2^ for women.

The baseline characteristics of the patient cohort stratified by sarcopenia status are shown in **Table 1**. Importantly, the sarcopenic and non-sarcopenic groups did not differ significantly in age, sex, race, smoking status, lung cancer histology, PDL-1 status, lung cancer stage or brain metastasis. Additionally, the percentage of irAE was similar in sarcopenic and non-sarcopenic patients. However, compared with patients who were not sarcopenic, a larger proportion of patients with sarcopenia had a higher ECOG performance score (P = 0.0395). For instance, 33.3% of sarcopenic patients compared to 14.7% of non-sarcopenic patient had an ECOG performance score of 2, representing increased physical disability in the sarcopenic group. Overall, the PMI was significantly lower in patients with a higher ECOG performance score (**Figure 1C**, P = 0.0189). Additionally, a lower BMI was observed in sarcopenic compared to non-sarcopenic patients (median, 23.3 kg/m^2^ in sarcopenic patients vs. 26.9 kg/m^2^ in non-sarcopenic patients, P = 0.0014). Taken together, this data highlights that sarcopenic patients with advanced NSCLC have a lower baseline performance status in comparison to non-sarcopenic patients.

**Table 1:** Baseline characteristics stratified by sarcopenia status.

	Total (n = 92)	Sarcopenic (n = 24)	Non-Sarcopenic (n = 68)	P value
**Age**, median (range), year **Sex**, n (%)	64 (36-89)	66.5 (40-83)	64 (36-89)	0.7353
Male Female	48 (52.2%) 44 (47.8%)	12 (50%) 12 (50%)	36 (52.9%) 32 (47.1%)	0.8170
**Race**, n (%) White/Caucasian Black/African American Other	77 (83.7%) 14 (15.2%) 1 (1.1%)	20 (83.3%) 4 (16.7%) 0 (0%)	57 (83.8%) 10 (14.7%) 1 (1.5%)	0.8194
**ECOG**, n (%) 0 1 2	22 (23.9%) 52 (56.5%) 18 (19.6%)	2 (8.3%) 14 (58.3%) 8 (33.3%)	20 (29.4%) 38 (55.9%) 10 (14.7%)	0.0395
**Smoking status**, n (%) Never smoked Current or former smoker	13 (14.1%) 79 (85.9%)	2 (8.3%) 22 (91.7%)	11 (16.2%) 57 (83.8%)	0.5019
**Histology**, n (%) Adenocarcinoma Squamous cell carcinoma Other*	68 (73.9%) 17 (18.5%) 7 (7.6%)	18 (75.0%) 5 (20.8%) 1 (4.2%)	50 (73.5%) 12 (17.7%) 6 (8.8%)	0.7378
**PDL-1 expression**, n (%) <1% 1-49% >=50% Unknown	44 (47.8%) 20 (21.7%) 16 (17.4%) 12 (13.1%)	13 (54.2%) 6 (25.0%) 3 (12.5%) 2 (8.3%)	31 (45.6%) 14 (20.6%) 13 (19.1%) 10 (14.7%)	0.6983
**Stage**, n (%) Locally advanced Metastatic	9 (9.8%) 83 (90.2%)	0 (0%) 24 (100%)	9 (13.2%) 59 (86.8%)	0.1053
**Brain metastasis**, n (%) No Yes	58 (63.0%) 34 (37.0%)	13 (54.2%) 11 (45.8%)	45 (66.2%) 23 (33.8%)	0.3313
**Treatment received,** n (%) Carboplatin/Pemetrexed/Pembrolizumab Carboplatin/Paclitaxel/Pembrolizumab Carboplatin/Docetaxel/Pembrolizumab Atezolizumab/Bevacizumab/Carboplatin/Paclitaxel Atezolizumab/Bevacizumab/Carboplatin/Pemetrexed	59 (64.1%) 30 (32.6%) 1 (1.1%) 1 (1.1%) 1 (1.1%)	16 (66.7%) 7 (29.2%) 0 (0%) 1 (4.2%) 0 (0%)	43 (63.2%) 23 (33.8%) 1 (1.5%) 0 (0%) 1 (1.5%)	0.4496
**irAE**, n (%) 0 >1	49 (53.3%) 43 (46.7%)	12 (50%) 12 (50%)	37 (54.4%) 31 (45.6%)	0.8131
**BMI**, median (IQR), kg/m^2^ Male Female	25.5 (22.5-29.6) 24.7 (21.8-27.6) 27.4 (23.6-33.2)	23.3 (20.7-25.0) 22.1 (20.5-23.8) 24.9 (22.6-28.3)	26.9 (23.1-31.7) 26.6 (23.1-29.2) 30.4 (23.8-34.4)	0.0014 0.0010 0.0904
**Serum albumin**, median (IQR), g/dl Male Female	4.0 (3.7-4.3) 4.0 (3.6-4.3) 4.0 (3.7-4.3)	3.9 (3.6-4.2) 4.0 (3.6-4.2) 3.8 (3.6-4.0)	4.0 (3.7-4.3) 4.0 (3.6-4.3) 4.0 (3.8-4.3)	0.2009 0.8367 0.0765
**NLR**, median (IQR) Male Female	6.2 (3.2-9.6) 6.7 (4.1-9.6) 5.2 (2.9-11.7)	6.2 (4.0-9.1) 6.7 (4.9-9.1) 4.5 (2.8-10.0)	6.3 (3.1-10.1) 6.7 (3.4-9.6) 6.0 (3.0-13.0)	0.9244 0.8052 0.6342
**Prognostic nutritional index**, median (IQR) Male Female	46.3 (41-49.8) 46.1 (40.5-48.8) 46.5 (42.2-50.7)	45.9 (41.0-47.5) 46.1 (40.6-47.6) 45.7 (41.0-47.3)	46.7 (41.2-51.2) 45.8 (40.5-52.3) 47.3 (43.6-51.1)	0.1979 0.5367 0.2119
**Psoas muscle index**, median (IQR), cm^2^/m^2^ Male Female	6.69 (5.66-7.78) 7.41 (6.04-8.33) 6.13 (5.11-6.90)	5.03 (4.61-5.44) 5.44 (5.02-5.81) 4.81 (4.33-5.06)	7.33 (6.46-8.23) 7.78 (7.17-8.78) 6.67 (5.99-7.51)	<0.0001 <0.0001 <0.0001
**Intramuscular adipose content**, median (IQR) Male Female	-0.31 (-0.40 to -0.24) -0.33 (-0.44 to -0.25) -0.29 (-0.35 to -0.23)	-0.31 (-0.42 to -0.23) -0.36 (-0.44 to -0.24) -0.29 (-0.41 to -0.21)	-0.30 (-0.39 to -0.24) -0.33 (-0.44 to -0.26) -0.29 (-0.35 to -0.23)	0.9835 0.7222 0.6853

Sarcopenic and non-sarcopenic patients were compared. P values were calculated with the Mann-Whitney U test for categorical data sets, Fisher’s exact test and Pearson’s χ2 test for numerical data sets. PDL-1, Programmed death-ligand 1; ECOG PS, Eastern Cooperative Group performance status; BMI, body mass index; NLR, neutrophil-to-lymphocyte ratio; irAE, immune-related adverse event.

*Other histologies include poorly differentiated carcinoma not otherwise specified (n = 3), poorly differentiated carcinoma with sarcomatoid carcinoma (n = 2) and SMARCA4-deficient malignant neoplasm (n = 2).

### Difference in overall survival between sarcopenic and non-sarcopenic patients

The median follow-up after initiation of concurrent immune checkpoint inhibitor (ICI) and chemotherapy (CTX) was 29.6 months. The median OS was 17.8 months. Importantly, sarcopenia was associated with significantly lower OS (**Figure 1D**, median OS, 9.1 months in sarcopenic patients vs. 22.3 months in non-sarcopenic patients, P = 0.002). A subgroup analysis including only patients with metastatic disease (n = 83, 90.2%) revealed similar results (**Supplementary Figure 1**). In order to determine whether this difference in OS is specific to our psoas muscle area measurements or can be attributed to muscle composition and quality, we next assessed the intramuscular adipose content (IMAC). The IMAC, defined by the psoas muscle and fat attenuation ratio, differed significantly between males and females (**Figure 1E**, median, -0.33 for men and -0.29 for women, P = 0.0339). However, we did not observe a significant difference in IMAC comparing sarcopenic and non-sarcopenic patients (**Table 1**, median IMAC, -0.31 in sarcopenic and -0.30 in non-sarcopenic patients, P = 0.9835). Additionally, OS was similar between patient with a high and low/normal IMAC (**Figure 1F**, median OS, 17.3 months in patients with high IMAC vs. 18.2 months in patients with normal/low IMAC, P = 0.5407). To summarize, these findings suggest that sarcopenia, determined by the PMI, is predictive for OS in this patient cohort, whereas muscle composition and quality do not have the same predictive value.

### Association of BMI and PMI

**Figure 2:**
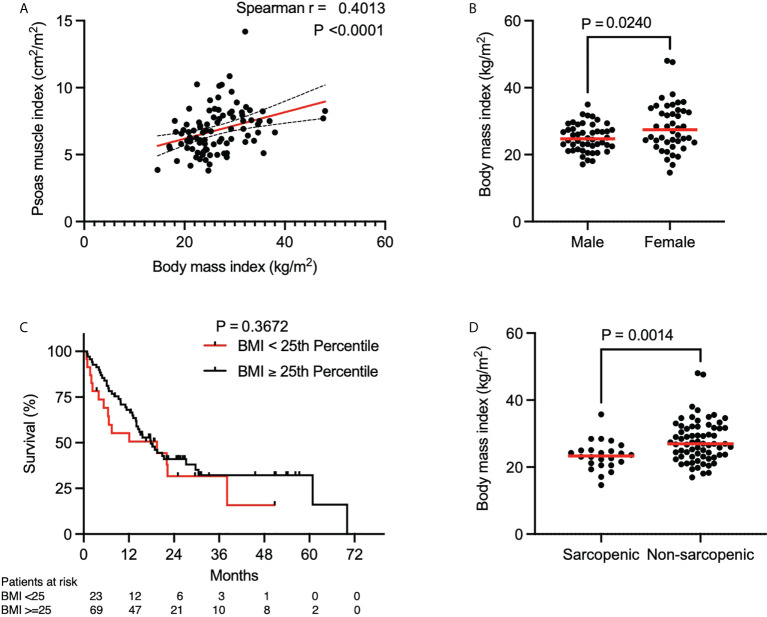
Association of psoas muscle index and body mass index. **(A)** Spearman correlation between BMI and PMI. Red line indicates the line of best fit. Dashed lines above and below the red line indicate the 95% confidence bands of the best fit line. **(B)** BMI stratified by gender (n = 92). P value calculated using the Mann-Whitney U test. **(C)** Kaplan-Meier curve depicting survival analysis based on BMI. P value calculated with the log-rank test. Each tick mark on the Kaplan-Meier curve represents a censored event. **(D)** BMI stratified by sarcopenia (n = 92). P value calculated using the Mann-Whitney U test. Each data point represents an individual patient. Red lines in **(B, D)** indicate the median of each dataset.

Since BMI is commonly used as a prognostic marker in clinical settings, we investigated the association of PMI and BMI. We found a weak linear association between increasing BMI and PMI (**Figure 2A**, Spearman r = 0.40, P < 0.0001). BMI was significantly different between males and females, but in contrast to PMI, BMI was higher in females than males (**Figure 2B**, median, 24.7 kg/m^2^ in males vs. 27.4 kg/m^2^ in females, P = 0.0240). Further analysis showed that the BMI differed significantly between sarcopenic and non-sarcopenic patients (**Figure 2D**, median, 23.3 kg/m^2^ vs 26.9 kg/m^2^, P = 0.0014). Of note, even with a significant lower BMI in the sarcopenic group, a majority of sarcopenic patients had a normal BMI (median 23.3 kg/m^2^, IQR 20.7-25.0 kg/m^2^). Consistent with the prior analysis of survival in sarcopenic and non-sarcopenic patients, the patient cohort was split into two groups: those with a BMI lower than the 25^th^ percentile and those with a BMI higher or equal to the 25^th^ percentile (21.8 kg/m^2^ for males and 23.6 kg/m^2^ for females). A difference in OS was not noted between these groups (**Figure 2C**, median OS, 19.5 months in patients with a BMI < 25^th^ percentile and 17.8 months in patients with a BMI ≥ 25^th^ percentile, P = 0.3672). These results suggest that BMI is not an adequate and reliable surrogate parameter for PMI as it does not predict OS in patients with advanced NSCLC treated with concurrent ICI and CTX.

### Multivariate analysis of overall survival

To further validate our results on sarcopenia determined by the PMI, we included age, gender, ECOG performance status, lung cancer histology, smoking status, BMI, neutrophil-to-lymphocyte ratio (NLR), prognostic nutritional index (PNI) and irAE in a multivariate Cox proportional hazards model. The results of the multivariate survival analysis in **Table 2** show that sarcopenia (HR 2.12, CI 1.10-3.97, P = 0.0209), ECOG >= 2 (HR 2.88, CI 1.42-5.71, P = 0.0027), PNI (HR 3.02, CI 1.42-6.26, P = 0.0034) and the absence of irAE (HR 2.04, CI 1.14-3.75, P = 0.0185) were independently associated with inferior survival in this cohort of patients with advanced lung cancer. The differences in median OS stratified by sarcopenia, PNI and irAE were 13.2 months (**Figure 1D**, P = 0.002), 12.8 months (**Supplementary Figure 2A**, P = 0.0036) and 15.6 months (**Supplementary Figure 2B**, P = 0.0072), respectively. In summary, this underlines that sarcopenia is an important and independent predictor of OS in patients with advanced NSCLC undergoing concurrent ICI and CTX.

**Table 2:** Cox proportional hazards regression model assessing the effect of patient-specific variables on overall survival.

	Cox proportional hazards regression (Wald test, P <0.0001)		
	HR	95% CI	P value
Age < 75 Male gender **ECOG >= 2** Squamous histology Never smoker BMI < 18.5 kg/m^2^ NLR < 3.24 **Sarcopenia** **PNI < 41** **irAE = 0**	0.72 0.98 **2.88** 0.73 0.49 0.81 1.28 **2.12** **3.02** **2.04**	0.36-1.55 0.55-1.75 **1.42-5.71** 0.32-1.49 0.16-1.21 0.25-2.24 0.62-2.52 **1.10-3.97** **1.42-6.26** **1.14-3.75**	0.3791 0.9454 **0.0027** 0.4195 0.1539 0.7100 0.4906 **0.0209** **0.0034** **0.0185**

Cox proportional hazards regression model: (Days, Survival) ~ Age < 75 + Male gender + ECOG >= 2 + Squamous histology + Never smoker + BMI < 18.5 + NLR < 3.24 + Sarcopenia + PNI < 41 + Number of irAE = 0. HR, hazard ratio; CI, confidence interval; ECOG PS, Eastern Cooperative Group performance status; BMI, body mass index; NLR, neutrophil-to-lymphocyte ratio; PNI, prognostic nutrition index; irAE, immune-related adverse event. Variables significantly associated with overall survival are highlighted in bold.

## Discussion

In this study, we evaluated the prognostic value of sarcopenia and its relation to PMI, ECOG and BMI in patients with advanced NSCLC undergoing concurrent ICI and CTX. We demonstrated that sarcopenia, defined by a PMI below the sex specific 25^th^ percentile, was associated with inferior OS. OS was 13.2 months shorter for patients with sarcopenia (P = 0.002). This association remained significant after adjusting for clinically relevant confounders such as age, gender, ECOG performance status, histology, smoking, BMI, NLR, PNI and irAE. We reported a hazard ratio of 2.12 (CI 1.1-3.97) for sarcopenia that is consistent with similar studies on sarcopenia and survival of patients with NSCLC by Tsukagoshi et al. (n = 30, HR 2.57, CI 1.02-6.46) (21) and Takada et al. (n = 103, HR 2.04, CI 1.14-3.63) (22). Shiroyama et al. investigated the impact of sarcopenia on progression-free survival in 42 patients with advanced NSCLC treated with PD-1 inhibitors and found that progression free survival was 2.1 months in patients with sarcopenia and 6.8 months in those without sarcopenia (14). Another study by Takahashi et al. included 315 patients with early-stage NSCLC and showed that sarcopenia is also associated with unfavorable postoperative complications and poor long-term survival (15). A larger study by Prado and colleagues analyzed the total skeletal muscle area on lumbar CT images of 2115 obese patients with solid cancers of the respiratory or gastrointestinal tract and found that sarcopenia was associated with increased mortality (HR 4.2, P < 0.0001) (16). Of note, the definition of sarcopenia and endpoints varied among these studies and, in contrast to our study, the patients included in these studies did not receive concurrent CTX and ICI (**Supplementary Table 1**). In our study we focused on overall survival as an endpoint which is patient-centered, objective and generally considered as the gold standard in clinical trials. The landmark trials on combined chemotherapy and immunotherapy all focused on overall survival as a primary endpoint and their data demonstrated a significant improvement in overall survival (17–19, 23). In our multivariate analysis we included primarily patient-specific factors such as age, gender, body mass index and performance status rather than disease-specific factors as these did not differ significantly between sarcopenic and non-sarcopenic patients.

Our study demonstrates that the PMI can reliably be assessed in lung cancer patients by analyzing cross sectional imaging from CT or PET/CT images obtained for diagnostic purposes prior to treatment initiation. Additionally, our findings highlight the importance of muscle mass rather than BMI for risk stratification. Specifically, we showed that a majority of sarcopenic patients had a normal BMI (**Figure 2B**, median 23.3 kg/m^2^, IQR 20.7-25.0 kg/m^2^) and we did not observe a significant difference in OS when solely stratifying by BMI. An apparently normal BMI may mask sarcopenia in patients with advanced lung cancer. These findings are consistent with a previous study that showed that muscle wasting is a prominent feature in a prospective cohort of 441 lung cancer patients despite a normal or increased BMI (24). Our findings suggest that BMI assessment is insufficient to identify sarcopenic patients with a high risk of poor OS. Therefore, we suggest that the assessment of skeletal muscle mass fundamental to the diagnosis of sarcopenia should be routinely included in NSCLC trials.

In our multivariate analysis, sarcopenia and ECOG performance status were both independently associated with reduced overall survival. This suggests that the objective assessment of sarcopenia by secondary analysis of cross-sectional imaging is important and complements the subjective assessment of patients by ECOG performance status. A substantial proportion (33%) of sarcopenic patients in our study had an ECOG performance status ≥ 2. This group of patients, frequently excluded from clinical trials examining concurrent CTX and ICI in NSCLC, may constitute the most vulnerable cohort of patients in terms of treatment-related toxicities (17, 25, 26). Other prognostic factors in patients with NSCLC are the neutrophil to lymphocyte ratio (NLR) and prognostic nutritional index (PNI). Kos et al. studied both in 138 patients with NSCLC and reported that a NLR ≥ 3.24 and PNI < 49.5 were markers of poor OS (27). Although we did not observe a significant difference in NLR and PNI between sarcopenic and non-sarcopenic patients (**Table 1**), our multivariate survival analysis suggested that the PNI is an independent marker of poor OS in patients with advanced NSCLC (median OS 6.7 months in patients with a PNI < 25^th^ percentile and 19.5 months in patients with a PNI ≥ 25^th^ percentile, P = 0.0036, **Supplementary Figure 2A**).

Taken together, our study adds to the emerging evidence that frailty, sarcopenia and malnutrition play an important role in lung cancer survival among newly diagnosed NSCLC patients receiving concurrent ICI and CTX (26, 28). Mechanisms by which sarcopenia confers increased risk of mortality and strategies to improve muscle mass and function in patients with advanced NSCLC such as protein supplementation, resistance exercises or therapeutics such as anamorelin warrants further research (29, 30).

### Strengths and limitations

In the present study, we used the PMI to assess skeletal muscle mass in patients with advanced NSCLC. The European Working Group on Sarcopenia in Older People (EWGSOP) published a consensus paper on definition and diagnosis of sarcopenia (11). They proposed a muscle mass of two standard deviations below healthy adults as an operational definition of sarcopenia (11). Validated cut-off values for PMI at the L3 lumbar vertebra have not been published for the US population to the best of our knowledge (7, 31), and we were unable to recruit a large reference cohort of healthy adults with cross-sectional abdominal imaging. Therefore, we used the 25^th^ percentile of the PMI as an unbiased approach to define sex-specific cut-offs to classify patients as those with sarcopenia. Compared to PMI cut-off values reported for Asian adults, the obtained values were similar for men (6.03 vs. 6.36 cm^2^/m^2^) but higher for women (5.11 vs. 3.92 cm^2^/m^2^) (14). We obtained our psoas muscle measurements by secondary analysis of CT and PET/CT images as these images were already available for diagnostic purposes. The limitations of our study are the retrospective, single center design, variability in the timing of imaging acquisition prior to treatment initiation and clinical challenges defining irAE by attributing toxicities to distinct therapies in patients receiving concurrent ICI and CTX. The strengths of our study are that our study was based on a well-defined cohort of NSCLC patients with a comprehensive dataset eligible for multivariate analysis with adjustments for key clinical confounders, long follow-up, blinding of the investigators performing the image analysis for clinical outcome and survival analysis showing a statistically significant and clinically relevant difference in OS between sarcopenic and non-sarcopenic patients.

## Conclusions

Sarcopenia is a predictor of reduced OS for patients with advanced NSCLC undergoing concurrent ICI and CTX irrespective of age, sex, BMI and functional status. The assessment of muscle mass is fundamental to the diagnosis of sarcopenia. The 25^th^ percentile of the PMI in our cohort allowed for an unbiased assessment of sarcopenia and should be applied and validated in larger NSCLC patient cohorts. Additional studies are needed to explore strategies to improve muscle mass and function in patients with advanced NSCLC.

## Data availability statement

The raw data supporting the conclusions of this article will be made available by the authors, without undue reservation.

## Ethics statement

The studies involving human participants were reviewed and approved by the University of Virginia Institutional Review Board for Health Science Research per protocols 18465 and 19083. Written informed consent for participation was not required for this study in accordance with the national legislation and the institutional requirements.

## Author contributions

FB and RH were responsible for the conception and design of the study. RH, RG, and LS recruited patients and established a database. FB, SM, and NW were involved in the collection and compilation of data. FB, SM, NW, CH, and AK were involved in the image analysis. FB, WN, and RH were responsible for analysis and interpretation of data. FB and RH wrote the manuscript.

## Funding

This study was supported by the University of Virginia Department of Internal Medicine Resident Research Grant.

## Acknowledgments

We thank Andrew Grainger for bioinformatic assistance with the image analysis.

## Conflict of interest

RG has the following conflicts of interest to declare: Consulting or Advisory Role of Daiichi Sankyo, AstraZeneca, BluePrint Medicines, Pfizer, Mirati, Sanofi, Ococyte, Jazz Pharmaceuticals, Janssen; Research Funding not related to the current project directly paid to the institution: Pfizer, Mirati, Daiichi Sankyo, Jounce Therapeutics, Helsinn, Bristol Myers Squibb, Merck, Janssen, RTI International, AstraZeneca, Alliance Foundation Trials, Takeda, Hoosier Cancer Research Network, ECOG-ACRIN, NCI.

RH has the following conflicts of interest to declare: Consulting or Advisory Role of Bristol-Myers Squibb/Ono Pharmaceutical; Research Funding not related to the current project directly paid to the institution: Merck Sharp & Dohme, AstraZeneca/MedImmune, Mirati Therapeutics, Lilly and Daiichi Sankyo.

The remaining authors declare that the research was conducted in the absence of any commercial or financial relationships that could be construed as a potential conflict of interest.

## Publisher’s note

All claims expressed in this article are solely those of the authors and do not necessarily represent those of their affiliated organizations, or those of the publisher, the editors and the reviewers. Any product that may be evaluated in this article, or claim that may be made by its manufacturer, is not guaranteed or endorsed by the publisher.

## Supplementary material

Supplementary Figure 1 Association of sarcopenia with overall survival in patients with metastatic NSCLC. Kaplan-Meier curve depicting survival analysis based on sarcopenia in patients with metastatic NSCLC (n = 83). P value was calculated with the log-rank test. Each tick mark on the Kaplan-Meier curve represents a censored event.

Supplementary Figure 2 Association of prognostic nutritional index (PNI) and immune related adverse events (irAE) with overall survival. Kaplan-Meier curves depicting survival analysis based on PNI **(A)** and irAE **(B)**. P values were calculated with the log-rank test. Each tick mark on the Kaplan-Meier curve represents a censored event.

Supplementary Table 1 Selected studies evaluating the impact of sarcopenia determined by the psoas muscle index on prognosis of non-small cell lung cancer patients.

## Abbreviations

BIA, bio-impedance analysis; BMI, body mass index; CI, confidence interval; CT, computed tomography; ECOG, Eastern Cooperative Oncology Group; EWGSOP, European Working Group on Sarcopenia in Older People; CTX, chemotherapy; DXA, Dual x-ray absorptiometry; HR, hazard ratio; HU, hounsfield units; ICI, immune checkpoint inhibitor; IMAC, intramuscular adipose tissue content; irAE, immune-related adverse event; IQR, interquartile range; MRI, magnetic resonance imaging; NLR, neutrophil-to-lymphocyte ratio; NSCLC, non-small cell lung cancer (NSCLC); OS, overall survival; PDL-1, programmed death ligand 1; PNI, prognostic nutritional index; PMI, psoas muscle index.

## References

[1] Walston J Hadley EC Ferrucci L Guralnik JM Newman AB Studenski SA. Research agenda for frailty in older adults: Toward a better understanding of physiology and etiology: Summary from the American geriatrics Society/National institute on aging research conference on frailty in older adults. J Am Geriatr Soc (2006) 54(6):991–1001. doi: 10.1111/j.1532-5415.2006.00745.x 16776798

[2] NCBI. Frailty MeSH unique ID: D000073496: National center for biotechnology information, U.S. National Library of Medicine (2018). Available at: https://www.ncbi.nlm.nih.gov/mesh/?term=frailty.

[3] Fried LP Tangen CM Walston J Newman AB Hirsch C Gottdiener J. Frailty in older adults: Evidence for a phenotype. J Gerontol A Biol Sci Med Sci (2001) 56(3):M146–56. doi: 10.1093/gerona/56.3.M146 11253156

[4] Ensrud KE Ewing SK Taylor BC Fink HA Cawthon PM Stone KL. Comparison of 2 frailty indexes for prediction of falls, disability, fractures, and death in older women. Arch Intern Med (2008) 168(4):382–9. doi: 10.1001/archinternmed.2007.113 18299493

[5] Morley JE Baumgartner RN Roubenoff R Mayer J Nair KS. Sarcopenia. J Lab Clin Med (2001) 137(4):231–43. doi: 10.1067/mlc.2001.113504 11283518

[6] Takenaka Y Oya R Takemoto N Inohara H. Predictive impact of sarcopenia in solid cancers treated with immune checkpoint inhibitors: A meta-analysis. J Cachexia Sarcopenia Muscle (2021) 12(5):1122–35. doi: 10.1002/jcsm.12755 PMC8517360 34337889

[7] Yang M Shen Y Tan L Li W. Prognostic value of sarcopenia in lung cancer: A systematic review and meta-analysis. Chest (2019) 156(1):101–11. doi: 10.1016/j.chest.2019.04.115 31128115

[8] Buentzel J Heinz J Bleckmann A Bauer C Röver C Bohnenberger H. Sarcopenia as prognostic factor in lung cancer patients: A systematic review and meta-analysis. Anticancer Res (2019) 39(9):4603–12. doi: 10.21873/anticanres.13640 31519557

[9] Pamoukdjian F Bouillet T Lévy V Soussan M Zelek L Paillaud E. Prevalence and predictive value of pre-therapeutic sarcopenia in cancer patients: A systematic review. Clin Nutr (2018) 37(4):1101–13. doi: 10.1016/j.clnu.2017.07.010 28734552

[10] Deng HY Chen ZJ Qiu XM Zhu DX Tang XJ Zhou Q. Sarcopenia and prognosis of advanced cancer patients receiving immune checkpoint inhibitors: A comprehensive systematic review and meta-analysis. Nutrition (2021) 90:111345. doi: 10.1016/j.nut.2021.111345 34166897

[11] Cruz-Jentoft AJ Baeyens JP Bauer JM Boirie Y Cederholm T Landi F. Sarcopenia: European consensus on definition and diagnosis: Report of the European working group on sarcopenia in older people. Age Ageing (2010) 39(4):412–23. doi: 10.1093/ageing/afq034 PMC2886201 20392703

[12] Fearon K Strasser F Anker SD Bosaeus I Bruera E Fainsinger RL. Definition and classification of cancer cachexia: An international consensus. Lancet Oncol (2011) 12(5):489–95. doi: 10.1016/S1470-2045(10)70218-7 21296615

[13] Recio-Boiles A Galeas JN Goldwasser B Sanchez K Man LMW Gentzler RD. Enhancing evaluation of sarcopenia in patients with non-small cell lung cancer (NSCLC) by assessing skeletal muscle index (SMI) at the first lumbar (L1) level on routine chest computed tomography (CT). Support Care Cancer (2018) 26(7):2353–9. doi: 10.1007/s00520-018-4051-2 PMC5984123 29417293

[14] Shiroyama T Nagatomo I Koyama S Hirata H Nishida S Miyake K. Impact of sarcopenia in patients with advanced non-small cell lung cancer treated with PD-1 inhibitors: A preliminary retrospective study. Sci Rep (2019) 9(1):2447. doi: 10.1038/s41598-019-39120-6 30792455 PMC6385253

[15] Takahashi Y Suzuki S Hamada K Nakada T Oya Y Sakakura N. Sarcopenia is poor risk for unfavorable short- and long-term outcomes in stage I non-small cell lung cancer. Ann Transl Med (2021) 9(4):325. doi: 10.21037/atm-20-4380 33708952 PMC7944314

[16] Prado CM Lieffers JR McCargar LJ Reiman T Sawyer MB Martin L. Prevalence and clinical implications of sarcopenic obesity in patients with solid tumours of the respiratory and gastrointestinal tracts: A population-based study. Lancet Oncol (2008) 9(7):629–35. doi: 10.1016/S1470-2045(08)70153-0 18539529

[17] Gandhi L Rodríguez-Abreu D Gadgeel S Esteban E Felip E De Angelis F. Pembrolizumab plus chemotherapy in metastatic non-Small-Cell lung cancer. N Engl J Med (2018) 378(22):2078–92. doi: 10.1056/NEJMoa1801005 29658856

[18] Socinski MA Jotte RM Cappuzzo F Orlandi F Stroyakovskiy D Nogami N. Atezolizumab for first-line treatment of metastatic nonsquamous NSCLC. New Engl J Med (2018) 378(24):2288–301. doi: 10.1056/NEJMoa1716948 29863955

[19] Paz-Ares L Luft A Vicente D Tafreshi A Gümüş M Mazières J. Pembrolizumab plus chemotherapy for squamous non–Small-Cell lung cancer. New Engl J Med (2018) 379(21):2040–51. doi: 10.1056/NEJMoa1810865 30280635

[20] Okumura S Kaido T Hamaguchi Y Fujimoto Y Kobayashi A Iida T. Impact of the preoperative quantity and quality of skeletal muscle on outcomes after resection of extrahepatic biliary malignancies. Surgery (2016) 159(3):821–33. doi: 10.1016/j.surg.2015.08.047 26603849

[21] Tsukagoshi M Yokobori T Yajima T Maeno T Shimizu K Mogi A. Skeletal muscle mass predicts the outcome of nivolumab treatment for non-small cell lung cancer. Med (Baltimore) (2020) 99(7):e19059. doi: 10.1097/MD.0000000000019059 PMC7035054 32049805

[22] Takada K Yoneshima Y Tanaka K Okamoto I Shimokawa M Wakasu S. Clinical impact of skeletal muscle area in patients with non-small cell lung cancer treated with anti-PD-1 inhibitors. J Cancer Res Clin Oncol (2020) 146(5):1217–25. doi: 10.1007/s00432-020-03146-5 PMC11804506 32025867

[23] Paz-Ares L Ciuleanu TE Cobo M Schenker M Zurawski B Menezes J. First-line nivolumab plus ipilimumab combined with two cycles of chemotherapy in patients with non-small-cell lung cancer (CheckMate 9LA): An international, randomised, open-label, phase 3 trial. Lancet Oncol (2021) 22(2):198–211. doi: 10.1016/S1470-2045(20)30641-0 33476593

[24] Baracos VE Reiman T Mourtzakis M Gioulbasanis I Antoun S. Body composition in patients with non-small cell lung cancer: A contemporary view of cancer cachexia with the use of computed tomography image analysis. Am J Clin Nutr (2010) 91(4):1133S–7S. doi: 10.3945/ajcn.2010.28608C 20164322

[25] Kakinuma K Tsuruoka H Morikawa K Furuya N Inoue T Miyazawa T. Differences in skeletal muscle loss caused by cytotoxic chemotherapy and molecular targeted therapy in patients with advanced non-small cell lung cancer. Thorac Cancer (2018) 9(1):99–104. doi: 10.1111/1759-7714.12545 29067769 PMC5754304

[26] Hirsch FR Scagliotti GV Mulshine JL Kwon R Curran WJ Jr. Wu YL. Lung cancer: current therapies and new targeted treatments. Lancet (2017) 389(10066):299–311. doi: 10.1016/S0140-6736(16)30958-8 27574741

[27] Kos FT Hocazade C Kos M Uncu D Karakas E Dogan M. Assessment of prognostic value of “Neutrophil to lymphocyte ratio” and “Prognostic nutritional index” as a sytemic inflammatory marker in non-small cell lung cancer. Asian Pac J Cancer Prev (2015) 16(9):3997–4002. doi: 10.7314/APJCP.2015.16.9.3997 25987075

[28] Baldessari C Guaitoli G Valoriani F Bonacini R Marcheselli R Reverberi L. Impact of body composition, nutritional and inflammatory status on outcome of non-small cell lung cancer patients treated with immunotherapy. Clin Nutr ESPEN (2021) 43:64–75. doi: 10.1016/j.clnesp.2021.02.017 34024567

[29] Temel JS Abernethy AP Currow DC Friend J Duus EM Yan Y. Anamorelin in patients with non-small-cell lung cancer and cachexia (ROMANA 1 and ROMANA 2): Results from two randomised, double-blind, phase 3 trials. Lancet Oncol (2016) 17(4):519–31. doi: 10.1016/S1470-2045(15)00558-6 26906526

[30] Argilés JM Busquets S Stemmler B López-Soriano FJ. Cachexia and sarcopenia: Mechanisms and potential targets for intervention. Curr Opin Pharmacol (2015) 22:100–6. doi: 10.1016/j.coph.2015.04.003 25974750

[31] Bahat G Turkmen BO Aliyev S Catikkas NM Bakir B Karan MA. Cut-off values of skeletal muscle index and psoas muscle index at L3 vertebra level by computerized tomography to assess low muscle mass. Clin Nutr (2021) 40(6):4360–5. doi: 10.1016/j.clnu.2021.01.010 33516603

