# Supplementary figures and images for: Association of sarcopenia with survival in advanced NSCLC patients receiving concurrent immunotherapy and chemotherapy
Source: Front Oncol. 2022 Sep 23;12:986236. doi: 10.3389/fonc.2022.986236 (PMC9539742; doi:10.3389/fonc.2022.986236)

Suppl. Figure 1

A

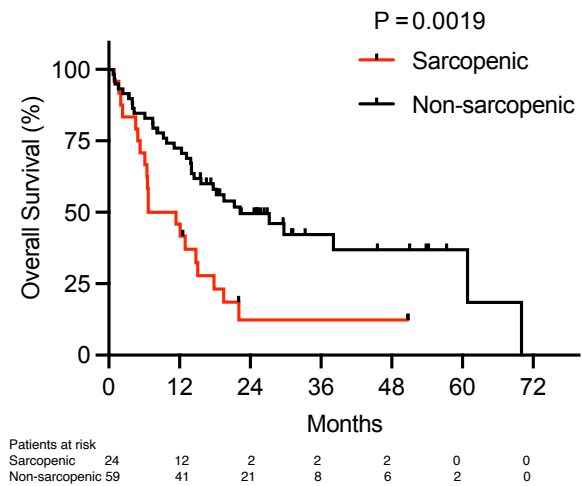

Suppl. Figure 2

A

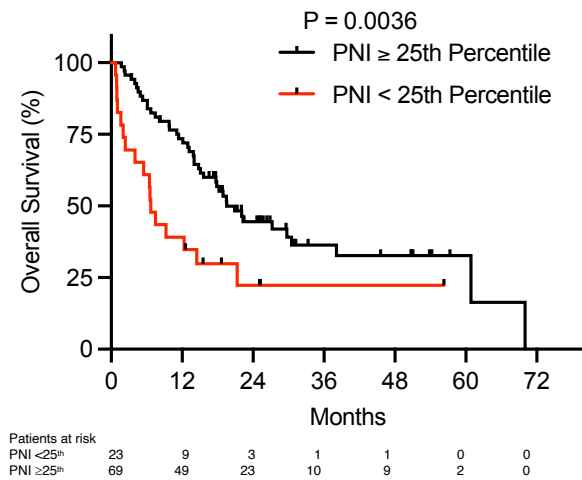

B

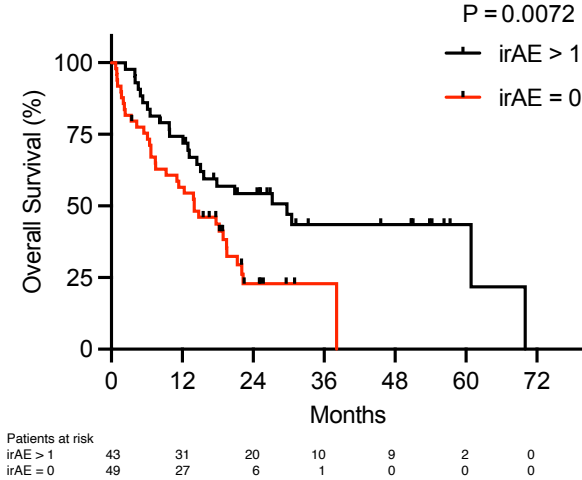

Supplement: Supplementary Figure 1 — Association of sarcopenia with overall survival in patients with metastatic NSCLC. Kaplan-Meier curve depicting survival analysis based on sarcopenia in patients with metastatic NSCLC (n = 83). P value was calculated with the log-rank test. Each tick mark on the Kaplan-Meier curve represents a censored event. [file Image_1.pdf]
