# Supplementary material for: Association of sarcopenia with survival in advanced NSCLC patients receiving concurrent immunotherapy and chemotherapy
Source: Front Oncol. 2022 Sep 23;12:986236. doi: 10.3389/fonc.2022.986236 (PMC9539742; doi:10.3389/fonc.2022.986236)
Supplement: Supplementary Table 1 — Selected studies evaluating the impact of sarcopenia determined by the psoas muscle index on prognosis of non-small cell lung cancer patients. [file Table_1.pdf]

Supplementary Table 1: Selected studies evaluating the impact of sarcopenia determined by the psoas muscle index on prognosis of non-small cell lung cancer patients

Author	Sarcopenia definition	Patient population	Endpoints
Tsukagoshi et al. (2020) (1)	Cross sectional area of the bilateral psoas muscles at the L3 level on CT scan divided by the height squared. Cut-off values were <3.92 cm^2/m^2 for women and <6.36 cm^2/m^2 for men.	30 patients with stage IIIB/IV NSCLC or recurrent NSCLC receiving nivolumab.	Progression free and overall survival
Takada et al. (2020) (2)	*Skeletal muscle area* at the L3 level on CT scan divided by the height squared. Cut-off values were <21.73 cm^2/m^2 for women and <25.63 cm^2/ m^2 for men.	103 patients with stage IIIB/IV NSCLC or recurrent NSCLC receiving either nivolumab or pembrolizumab.	Progression free and overall survival
Shiroyama et al. (2019) (3)	Cross sectional area of the bilateral psoas muscles at the L3 level on CT scan divided by the height squared. Cut-off values were <3.92 cm^2/m^2 for women and <6.36 cm^2/m^2 for men.	42 patients with advanced NSCLC receiving either nivolumab or pembrolizumab.	Progression free survival
Takahashi et al. (2021) (4)	Cross sectional area of the bilateral psoas muscles at the L3 level on CT scan. Cut-off values were 460.55 mm^2 for women and 491.53 mm^2 for men.	315 patients with stage I NSCLC who had undergone lobectomy with systematic nodal dissection.	Postoperative complications, recurrence-free survival and 5-year overall survival
Nakamura et. al. (2018) (5)	Cross sectional area of the bilateral psoas muscles at the L3 level on CT scan divided by the height squared. Cut-off values were <3.92 cm^2/m^2 for women and <6.36 cm^2/m^2 for men.	328 patients with stage I-III NSCLC who had undergone surgery	Overall survival

References

1. Tsukagoshi M, Yokobori T, Yajima T, Maeno T, Shimizu K, Mogi A, et al. Skeletal muscle mass predicts the outcome of nivolumab treatment for non-small cell lung cancer. Medicine (Baltimore). 2020;99(7):e19059.
2. Takada K, Yoneshima Y, Tanaka K, Okamoto I, Shimokawa M, Wakasu S, et al. Clinical impact of skeletal muscle area in patients with non-small cell lung cancer treated with anti-PD-1 inhibitors. J Cancer Res Clin Oncol. 2020;146(5):1217-25.
3. Shiroyama T, Nagatomo I, Koyama S, Hirata H, Nishida S, Miyake K, et al. Impact of sarcopenia in patients with advanced non-small cell lung cancer treated with PD-1 inhibitors: A preliminary retrospective study. Sci Rep. 2019;9(1):2447.
4. Takahashi Y, Suzuki S, Hamada K, Nakada T, Oya Y, Sakakura N, et al. Sarcopenia is poor risk for unfavorable short- and long-term outcomes in stage I non-small cell lung cancer. Ann Transl Med. 2021;9(4):325.
5. Nakamura R, Inage Y, Tobita R, Yoneyama S, Numata T, Ota K, et al. Sarcopenia in Resected NSCLC: Effect on Postoperative Outcomes. Journal of Thoracic Oncology. 2018;13(7):895-903.
